# Activation/Inhibition of mast cells by supra-optimal antigen concentrations

**DOI:** 10.1186/1478-811X-11-7

**Published:** 2013-01-22

**Authors:** Michael Huber

**Affiliations:** 1 Institute of Biochemistry and Molecular Immunology, University Clinic, RWTH Aachen University, Pauwelsstr. 30, 52074, Aachen, Germany

**Keywords:** Mast cell, Allergy, SHIP1, Lyn, PKC-δ, High-affinity receptor for IgE, Dose-response behavior, Inhibitory signalosome

## Abstract

Mast cells (MCs) are tissue resident cells of hemopoietic origin and are critically involved in allergic diseases. MCs bind IgE by means of their high-affinity receptor for IgE (FcεRI). The FcεRI belongs to a family of multi-chain immune recognition receptors and is activated by cross-linking in response to multivalent antigens (Ags)/allergens. Activation of the FcεRI results in immediate release of preformed granular substances (e.g. histamine, heparin, and proteases), generation of arachidonic acid metabolites, and production of pro-inflammatory cytokines. The FcεRI shows a remarkable, bell-shaped dose-response behavior with weak induction of effector responses at both low and high (so-called supra-optimal) Ag concentrations. This is significantly different from many other receptors, which reach a plateau phase in response to high ligand concentrations. To explain this unusual dose-response behavior of the FcεRI, scientists in the past have drawn parallels to so-called precipitin curves resulting from titration of Ag against a fixed concentration of antibody (Ab) in solution (a.k.a. Heidelberger curves). Thus, for high, supra-optimal Ag concentrations one could assume that every IgE-bound FcεRI formed a monovalent complex with “its own Ag”, thus resulting in marginal induction of effector functions due to absence of receptor cross-linking. However, this was never proven to be the case. More recently, careful studies of FcεRI activation and signaling events in MCs in response to supra-optimal Ag concentrations have suggested a molecular explanation for the descending part of this bell-shaped curve. It is obvious now that extensive FcεRI/IgE/Ag clusters are formed and inhibitory molecules and signalosomes are engaged in response to supra-optimal cross-linking (amongst them the Src family kinase Lyn and the inositol-5′-phosphatase SHIP1) and they actively down-regulate MC effector responses. Thus, the analysis of MC signaling triggered by supra-optimal crosslinking holds great potential for identifying novel targets for pharmacologic therapeutic intervention to benefit patients with acute and chronic allergic diseases.

## Lay abstract

Mast cells are central players in allergic diseases like hay fever and asthma. When allergens bind to specific receptors (called FcεRI) on the surface of mast cells, various intracellular events are triggered, resulting in the secretion of inflammatory mediators, such as histamine and proteases. These mediators are responsible for the well known symptoms of allergies (e.g. itching, sneezing, and coughing). In principle, receptors for different ligands are expressed by every cell, enabling each cell to sense its environment and react to it in an appropriate fashion. Usually, the concentration of the ligand correlates with the extent of the ligand-induced cellular response. Intriguingly, this is not the case for the FcεRI. High, so-called supra-optimal allergen concentrations result in only weak activation of mast cells, whereas lower concentrations of allergen can cause strong activation. The reason is that high allergen concentrations engage inhibitory mechanisms, which do not allow for the induction of mediator secretion. In recent years, several intracellular proteins involved in such inhibitory mechanisms have been identified and their functional interactions are currently being elucidated. Thus, further investigation of this cell-intrinsic shut-off mechanism and identification of additional proteins involved should enable researchers to define novel molecular targets for pharmacologic intervention. The aim would be to exploit this knowledge to actively silence the mast cell during pollen season.

## Review

### The cell and its receptor

Mast cells (MCs) are hemopoietically derived tissue resident cells that are concentrated in tissues close to the external environment, i.e., the skin and mucosal membranes of the intestine and airways [1]. One of their main functions is to act as guards, alerting the body to invasion by bacteria, parasites and viruses and initiating an inflammatory response [2-5]. On the pathophysiological side, amongst others MCs have attained inglorious publicity as central effector cells in acute allergic disorders [6]. Here, MCs recognize multivalent allergens/antigens (Ags) via IgE immunoglobulins, which are bound to high-affinity IgE receptors (FcεRI) on the surface of the MCs. Ag-triggered activation of the FcεRI then results in several pro-inflammatory responses, such as release of preformed mediators (e.g. histamine, proteoglycans, and proteases) from intracellular granules in a process called degranulation, and de-novo production and release of arachidonic acid metabolites (e.g. leukotrienes and prostaglandins) as well as cytokines and chemokines (e.g. IL-6, TNF-alpha and MCP-1) [7]. As a result, MCs play a central role in the development of type I hypersensitivity reactions [8,9].

**Figure 1:**
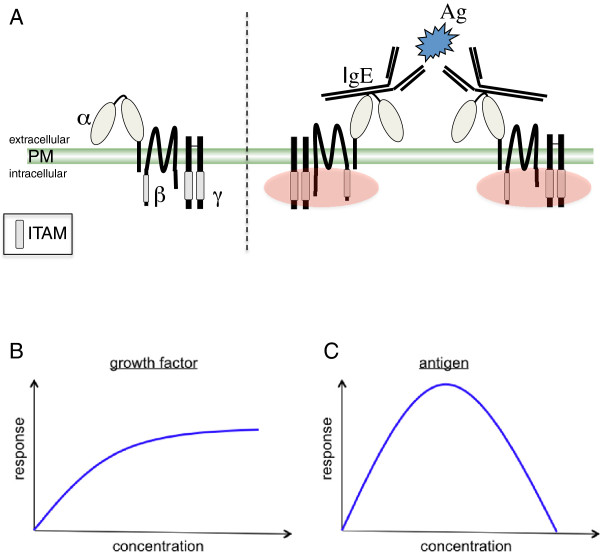
**The FcεRI and its dose**-**response behavior.** (**A**) The murine FcεRI consists of an α-, a β-, and two disulfide-bridged γ-subunits. The α-subunit contains only a short cytoplasmic region and binds to the constant Cε3 region of IgE via its second extracellular immunoglobulin-like domain (oval shape). The β-subunit belongs to the tetraspanin family and the γ-subunits can be called small transmembrane adaptor proteins. Due to its association with further activating Fc receptors the γ-subunit is now called FcRgamma. β-subunit and FcRgamma contain ITAMs in their cytoplasmic regions. The ITAMs are phosphorylated by Lyn (visualized by red ovals) subsequent to receptor crosslinking by multivalent Ag. Lyn has been shown to be pre-associated with the FcεRI. PM, plasma membrane. (**B**) Stimulation of growth factor receptors with increasing amounts of respective ligands usually results in a saturation of the response at high ligand concentrations. (**C**) The dose–response curve of the FcεRI traverses through three distinct phases. At low Ag concentrations, i. e. a high Ab/Ag ratio, only a weak response can be measured, at medium Ag concentrations (the zone of equivalence between Ab and Ag) an optimal response can be observed, and at high Ag concentrations, i. e. a low Ab/Ag ratio, only a weak response can again be measured. Therefore, this dose–response curve is called bell-shaped.

The FcεRI on murine MCs and basophils consists of an α-subunit, a β-subunit, and two disulfide-bridged γ-subunits (αβγ_2_) [10] (Figure 1A). The α-subunit, which contains only a short cytoplasmic tail of 17 amino acids, binds to the constant Cε3 region of the IgE molecule via its second extracellular immunoglobulin-like domain [11]. The β-subunit, a tetraspanin, and the γ-subunits contain immunoreceptor tyrosine-based activation motifs (ITAMs) in their cytoplasmic domains [12], which, when phosphorylated by the Src family kinase (SFK) Lyn after receptor crosslinking by multivalent Ag, trigger signaling events by attracting cytoplasmic proteins that contain phosphotyrosine-binding SH2-domains [7]. Lyn has been shown to be pre-associated with the FcεRI [13,14]. Of note, the γ-subunit of the FcεRI also associates with other activating Fc receptors and therefore is now referred to as FcRgamma [15]. Though the IgE Fc-fragment is dimeric by nature, IgE-induced crosslinking of FcεRIs does not occur in the absence of Ag. Interaction of IgE with the α-subunit of one FcεRI prohibits concomitant binding to a second FcεRI α-subunit [16]. Moreover, extensive glycosylation of the FcεRI α-subunit is thought to suppress spontaneous aggregation of the FcεRI [7]. Thus, activation requires crosslinking of two or more IgE-bound receptors by multivalent Ag. However, binding of IgE to the FcεRI is not a mere passive presensitization step to confer Ag specificity on the MC, but rather it induces Ag-independent signaling events that actively promote MC survival [17-19]. For completeness, it has to be added that whereas the FcεRI on murine MCs always contains the β-chain [10], the FcεRI can be expressed in two forms in human MCs and basophils, one containing the β-chain and one lacking the β-chain [11]. The β-chain-containing FcεRI is also expressed on human eosinophils, whereas murine eosinophils do not express FcεRI [20,21]. Moreover, the β-chain-lacking FcεRI can also be found in human monocytes, Langerhans cells and dendritic cells [22,23]. The exclusive expression of the β-chain-containing FcεRI in murine cells is because all three chains have to be present for cell surface expression [10]. In human cells, expression of the β-chain is dispensable for surface expression of the FcεRI, however, the human β-chain has been shown by Kinet and colleagues to exert two important amplifier functions. Compared to a human FcεRI that only consists of α-chain and FcRgamma, the presence of the β-chain enhances both FcεRI stability and surface expression as well as activation mechanisms [24,25].

### Cross-link it – the precipitin way?

**Figure 2:**
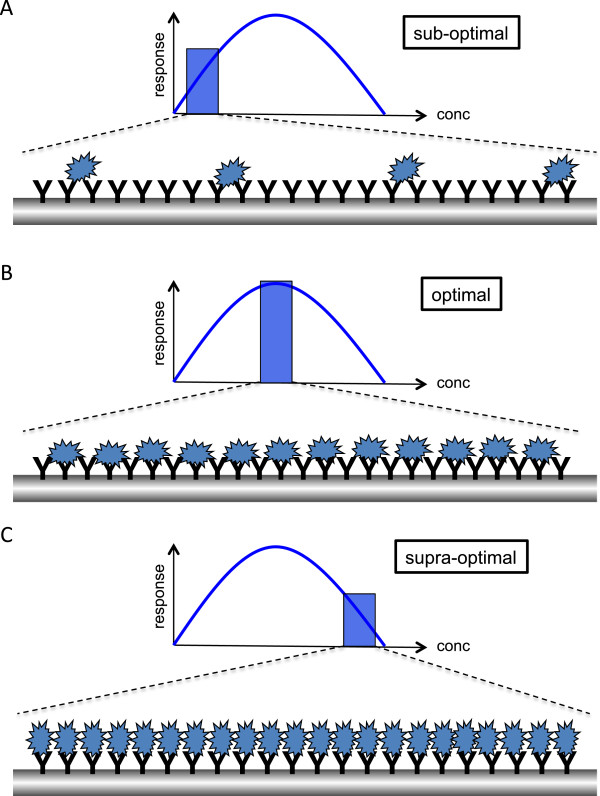
**The precipitin-type model-a first attempt to explain FcεRI dose-response behavior.** (**A**) A sub-optimal dose of multivalent Ag would only cross-link a minor fraction of IgE-bound FcεRIs and result in only weak downstream signaling and induction of effector functions. (**B**) An equivalent Ag concentration optimally would cross-link all IgE-bound receptors and cause maximal activation of Ag-triggered signaling pathways and effector functions. (**C**) High, supra-optimal concentrations of multivalent Ag were initially thought to result in the formation of monovalent complexes with every IgE binding “its own” Ag. Thus, cross-linking of IgE-bound FcεRI should not occur. Comparable to the sub-optimal situation only weak signaling and execution of effector functions would be the outcome. Of note, this model of FcεRI activation based on the precipitin curve of in-solution immunoprecipitation is not supported by experimental data (see text).

Many receptors, like receptor tyrosine kinases and cytokine receptors, require ligand-mediated dimerization for their activation [26]. The dose-response behavior of such receptors differs significantly from that of the FcεRI. These different dose-response curves are sketched in Figure 1. Whereas increasing concentrations of growth factors result in a saturated response (e.g. production of cytokines or upregulation of cell surface molecules), the Ag dose-response curve of the FcεRI is a bell-shaped one showing weak responses at both low and high Ag concentrations (e.g. degranulation and proinflammatory cytokine production) [27,28]. Such a curve is reminiscent of the so-called precipitin curves resulting from the titration of Ag against a fixed amount of antiserum in solution (a.k.a. Heidelberger curves) [29]. In such an assay, only an optimal ratio between Ag and Ab (called the zone of equivalence) results in the formation of a visible precipitate, whereas both excess Ab or surplus Ag prevents formation of a visible precipitate. In the typical laboratory situation, MCs are preloaded with IgE (usually of one specificity, such as anti-dinitrophenyl (DNP) IgE) and then are stimulated with different concentrations of the respective multivalent Ag, such as DNP_30-40_-HSA [27]. Alternatively, cross-linking can be induced by using anti-IgE immunoglobulins. Because of the similarities between the Ag/IgE-induced bell-shaped dose-response curves obtained with MCs and basophils and the in-solution precipitin curves, interpretation of the bell-shaped Ag/IgE-induced dose-response curve was often as sketched in Figure 2. Specifically, a sub-optimal dose of multivalent Ag (i. e. a high Ab/Ag ratio) was thought to cross-link only a few IgE-bound FcεRIs, resulting in a weak activation of downstream signaling pathways and effector functions (Figure 2A). An optimal Ag concentration, reflecting the zone of equivalence, was thought to optimally cross-link all IgE-bound receptors, thus causing strong activation of Ag-triggered signaling pathways and maximal induction of effector functions (Figure 2B). Increasing the Ag dose to the so-called supra-optimal concentration range (i. e. a low Ab/Ag ratio) was then thought to result in the formation of monovalent complexes with every IgE binding “its own” Ag and thus Ag-mediated cross-links between different IgE-bound FcεRIs were lost (Figure 2C). Similar to the sub-optimal situation, only marginal execution of effector functions combined with weak activation of downstream signaling pathways would thus be attained.

### The precipitin-type model of FcεRI regulation revisited

In 1973, Becker et al. investigated IgE/FcεRI redistribution and histamine release in human basophils in response to increasing concentrations of anti-IgE Abs [30]. They found that histamine release only occurred in the presence of sub-optimal to optimal levels of anti-IgE, whereas IgE/FcεRI redistribution was mainly observable at higher, supra-optimal concentrations of cross-linking agent [30]. This interesting finding was re-addressed by Magro and Alexander using a different approach [31]. In their experiments they studied histamine release from leukocytes of ragweed sensitive donors, again using anti-IgE Abs to cross-link IgE-bound FcεRIs. They were interested in the effects of monomeric Fab fragments of these anti-IgE Abs and found that for a sub-optimal to optimal concentration of anti-IgE increasing concentrations of the monomeric Fab fragments inhibited the release of histamine. This was expected since the monomers reduced receptor cross-linking by the intact anti-IgE Abs. At supra-optimal concentrations of anti-IgE Abs, however, increasing concentrations of the monomer enhanced histamine release. Thus, they concluded that the descending portion of the dose-response curve might be the result of an active turn-off mechanism caused by an excess of bridging rather than the reduced bridging due to monovalent interactions depicted in the model in Figure 2C [31]. Of note, the model shown in Figure 2 was only based on similarity to the Heidelberger curves of in-solution immunoprecipitation and not on experimental data.

How would secretion of histamine be suppressed when FcεRIs are supra-optimally cross-linked? Baird and coworkers reported that stimulation of IgE-bound FcεRI by polyclonal Abs against IgE induced a detergent-resistant association of these complexes with the cellular cytoskeleton [32]. In thorough dose-response studies, the extent of the cytoskeletal association followed the extent of FcεRI bridging and, more importantly, continued to increase beyond the anti-IgE concentration where degranulation was maximal [32]. This strongly indicated that stimulation of MCs with supra-optimal Ag concentrations, that results in little or no degranulation, did cause intracellular rearrangements. A further study by Oliver and colleagues verified that detergent-insolubility of FcεRI-IgE-Ag complexes did not correlate with degranulation [33]. Moreover, using inhibitors of actin polymerization, they could increase degranulation at supra-optimal Ag concentrations, while detergent-insolubility was decreased [33]. From these studies they concluded that Ag-triggered actin polymerization is more likely related to the termination/inhibition than to the stimulation of degranulation, in particular at supra-optimal Ag concentrations [33]. This also suggested that stronger actin polymerization was induced by supra-optimal than by optimal doses of Ag. It further indicated that strong activation of signal transduction events, at least those related to actin polymerization, occurred at supra-optimal Ag concentrations. Finally, Seagrave et al. using scanning electron microscopy showed that optimally cross-linked FcεRIs redistribute into chains and small clusters, whereas supra-optimally cross-linked receptors redistribute into clusters and large aggregates [34]. Again, large aggregates at high stimulus concentrations were prevented from forming by adding either monovalent ligands or actin depolymerizing agents.

Thus, suppressed degranulation in response to supra-optimal Ag appeared not to be due to reduced FcεRI crosslinking, as depicted in Figure 2C. This then begged the question, was the optimal crosslinking of the majority of receptors shown in Figure 2B accurate? To partially address this it was demonstrated, using stable oligomers of IgE prepared by chemical crosslinking, that no more than a few hundred trimers or an even smaller number of higher oligomers are required to induce a considerable amount of degranulation from RBL-2H3 MCs [35]. Using monoclonal Abs directed against the α-chain of the FcεRI, a study by Pecht and colleagues not only demonstrated that FcεRI aggregates as small as dimers are capable of providing an effective stimulus for degranulation but that only a small fraction of FcεRI needs to be cross-linked in order to yield optimal degranulation [36]. Also, for basophils, MacGlashan showed that only a few hundred FcεRI are needed for meaningful secretion to occur [37].

### Signal transduction in the supra-optimal world – an inhibitory signalosome?

All these studies indicated that the bell-shaped dose-response curve of MC degranulation might not reflect the level of signaling taking place inside the cells. Thus, it was important to elucidate the signaling pathways controlling MC activation in response to differing concentrations of Ag. A critical step in regulating IgE-induced degranulation is the release of intracellular Ca^2+^ ions from the ER with subsequent influx of extracellular Ca^2+^ via store-operated Ca^2+^ (SOC) channels [7,38,39]. This response was shown to follow the bell-shaped dose-response curve with weak Ca^2+^ mobilization in response to sub- as well as supra-optimal Ag concentrations and strong responses after optimal cross-linking [27]. Interestingly, Ca^2+^ mobilization at supra-optimal was not simply weaker than at optimal Ag concentrations, but the sustained pattern (optimal) changed to a transient one (supra-optimal), suggesting active negative regulation. Consistent with this, early overall tyrosine phosphorylation events were slightly enhanced in supra-optimally vs. optimally stimulated MCs [27,40]. Particularly, tyrosine phosphorylation of a 145 kDa protein was increased in parallel to the Ag levels and this protein was identified as the SH2-containing inositolpolyphosphate 5′-phosphatase, SHIP1, a critical negative regulator of PI3K signaling [27,41,42]. SHIP1 hydrolyzes phosphatidylinositol-3,4,5-trisphosphate (PIP_3_), the product of the PI3K-catalyzed reaction, to yield PI-3,4-P_2_[43,44]. This indicated that SHIP1 was involved in the regulation of the descending part of the dose-response curve and, indeed, SHIP1-deficient bone marrow-derived MCs (BMMCs) only showed weak or no reduction of degranulation in response to supra-optimal antigen concentrations. Correlating with this, SHIP1-deficient BMMCs, even at supra-optimal conditions, showed sustained Ca^2+^ mobilization [27].

Related to this, the SFK Lyn has been shown to phosphorylate and activate SHIP1 in MCs and degranulation studies with Lyn−/− BMMCs have revealed that these cells do not display the descending part of the bell-shaped degranulation curve [45]. Interestingly, Lyn has also been shown to tyrosine phosphorylate protein kinase C-δ (PKC-δ) and complexes of Lyn with SHIP1 and PKC-δ have been reported [46-48]. Relevant to this, Leitges et al. observed augmented Ag-triggered degranulation in PKC-δ-deficient BMMCs, in particular in response to supra-optimal stimulus concentrations [47]. These data strongly suggest the existence of an inhibitory signalosome, which appears to be particularly active when MCs are stimulated by supra-optimal Ag concentrations.

How could one envision the functional coupling between supra-optimally cross-linked FcεRIs and such an inhibitory signalosome? Intriguingly, the β-subunit of the FcεRI, which has been carefully described by Kinet and colleagues as the amplifier of FcεRI-mediated activation signals [25,49], has been found by different laboratories to be most highly tyrosine-phosphorylated in response to high Ag concentrations [27,50,51]. In this respect, in a very elegant and thorough study, Xiao et al. demonstrated that Lyn was most activated under supra-optimal conditions, resulting in pronounced β-subunit ITAM as well as SHIP1 tyrosine phosphorylation and suppressed degranulation as well as cytokine production [51]. Importantly, there are two differences between the β-chain and FcRgamma ITAMs, namely the β-ITAM has a shorter spacer between its two YXXL sequences (six amino acids (β) vs. seven amino acids (FcRgamma)), and the spacer region of the β-chain contains an additional tyrosine residue. Thus, the β-subunit via its unique ITAM may utilize Lyn to negatively regulate downstream events in response to supra-optimal Ag concentrations [51]. This puts the FcεRI β-chain and the SFK Lyn at the center of initiation of suppressive FcεRI-mediated signaling and SHIP1, by controlling the PI3K pathway, as one of the important downstream regulators of this response. However, there are certainly more signaling proteins involved in this process, such as PKC-δ mentioned above [47].

Interestingly, analyzing GPVI-mediated dense granule secretion from platelets, Kunapuli and coworkers found that functional interactions between Lyn, PKC-δ, and SHIP1 negatively regulate this process as well [52]. GPVI is a receptor for collagen on platelets that triggers signaling via an associated FcRgamma [53]. Since no FcεRI β-chain interacts with this receptor complex, the inhibitory signalosome comprising Lyn, PKC-δ, and SHIP1 appears to be capable of acting in a β-chain ITAM-independent manner. This is intriguing since the unique tyrosine residue between the canonical ITAM tyrosines of the β-chain has been shown to be very important for interaction with SHIP1 [54]. Nevertheless, this suggests that such an inhibiting/attenuating mechanism can also be functional in cells which only express an FcεRI lacking the β-chain, such as Langerhans and dendritic cells [22].

In MCs, two additional prominent PIP_3_ phosphatases are expressed that participate in regulation of the PI3K pathway, namely the 5′-phosphatase SHIP2 and the 3′-phosphatase PTEN. SHIP2 has been knocked-down by shRNA in BMMCs and shown to result in stronger FcεRI-induced degranulation at every Ag concentration tested, compared to cells treated with control shRNA [55]. However, the effect was most pronounced at optimal Ag concentrations and only marginal at supra-optimal Ag concentrations. Also, in contrast to SHIP1-deficient cells, there was no effect of SHIP2 reduction on Ag-triggered Ca^2+^ mobilization [55]. On the other hand, SHIP2 knock-down resulted in enhanced microtubule reorganization, which might explain the observed positive effect on degranulation in the absence of augmented Ca^2+^ mobilization [56]. These results indicate that SHIP1 is more involved than SHIP2 in repressing supra-optimal Ag-induced degranulation. PTEN, a prominent tumor suppressor, was also knocked-down by an shRNA approach in human MCs and cells with reduced PTEN expression were shown to react with augmented Ca^2+^ mobilization as well as degranulation in response to FcεRI activation [57]. Unfortunately, titration of cross-linking Ag was not extended to the supra-optimal range and thus, no statement can be made at present on PTEN’s role in repressing degranulation at very high Ag concentrations. However, this analysis did show that PTEN (and not SHIP1 nor SHIP2) is crucially involved in the “homeostatic” control of PIP_3_ levels in unstimulated MCs [57].

How SHIP1 controls degranulation in response to supra-optimal Ag concentrations is not entirely clear at the moment. Because of its structure, SHIP1 incorporates catalytic as well as adaptor functions [58]. Even on the basis of its catalytic activity two mechanisms, not necessarily mutually exclusive, could be responsible. First, by hydrolyzing PIP_3_, SHIP1 would suppress various PIP_3_-dependent molecules/pathways, one or more of them critically involved in Ca^2+^ mobilization and degranulation (e.g. phospholipase C-γ1 (PLC-γ1), PLC-γ2, and the tyrosine kinase Btk [7,59]). Second, by hydrolyzing PIP_3_, PI-3,4-P_2_ is generated, which is known to specifically interact with PH-domains of certain signaling proteins, e.g. the adaptor proteins Bam32/DAPP1, TAPP1, and TAPP2 [60]. Related to this, Bam32-deficiency has been demonstrated to result in augmented Ca^2+^ mobilization and degranulation in response to supra-optimal Ag concentrations, suggesting that Bam32 represents an effector of SHIP1’s negative activity [61]. Intriguingly, compared to wild-type BMMCs Bam32-deficient BMMCs show reduced Lyn and SHIP1 phosphorylation in response to Ag, indicating the presence of so far uncharacterized feed-back mechanisms [61].

With respect to SHIP1’s adaptor or scaffolding function, increasing numbers of interaction partners of SHIP1 have been and continue to be identified. SHIP1 contains an N-terminal SH2-domain, a centrally located 5′-phosphatase domain, and a C-terminus containing several proline-rich sequences as well as two NPxY motifs [58]. The SH2-domain is capable of binding to the phosphorylated ITAM sequences of the FcεRI β-chain and FcRgamma [62,63]. In keeping with this, additional ITAM sequences have been demonstrated to be bound by the SHIP1 SH2-domain [64-66]. Three of the proline-rich motifs in SHIP1’s C-terminus show good consensus for binding to SH3-domains of other proteins and several have been experimentally verified, such as Grb2, CIN85, and Src kinase [67-69]. This might suggest that Lyn via its SH3-domain can also interact with SHIP1 in the context of the inhibitory signalosome. Indeed, SHIP1 could be pulled-down from MC lysates by means of a GST-SH3 (Lyn) fusion protein (unpublished data). Recently, a pleckstrin homology-related domain has been identified that is present N-terminal to the 5′-phosphatase domain and shown to mediate membrane localization of SHIP1 by binding to PIP_3_[70].

Finally, proteins containing so-called phosphotyrosine-binding (PTB)-domains, such as the adaptor proteins Shc and p62Dok1, have been demonstrated to bind to SHIP1’s C-terminal NPxY motifs upon their phosphorylation [71-73]. Since Shc contains both a PTB-domain as well as an SH2-domain, it was thought initially that Shc took SHIP1 to the FcεRI. However, analysis of SHIP1-deficient BMMCs suggested a different binding order since FcεRI-mediated Shc tyrosine phosphorylation was dependent on SHIP1 expression [74]. Thus, Shc might be involved in limiting SHIP1’s activity at the receptor by “tearing” it away upon Shc’s tyrosine phosphorylation by Lyn. Shc, however, has also been implicated as a linker between SHIP1 and PKC-δ by binding to SHIP1 via its PTB-domain and to PKC-δ by means of its SH2-domain [47]. The adaptor protein p62Dok1, a well-known interaction partner of the GTPase-activating protein RasGAP, has also been shown to bind to SHIP1 and inhibit p21Ras and hence, be a negative regulator of the canonical MAPK pathway (Erk1/2) [72,73]. Interestingly, Erk1/2 might positively regulate FcεRI-mediated MC degranulation via two different pathways. Pecht and colleagues have reported that Erk1 is part of a feed-forward loop positively controlling Syk activity upon FcεRI triggering [75]. Pharmacologic inhibition of the Erk kinase MEK suppressed Ag-induced MC degranulation [75]. This effect was recently corroborated with novel MEK inhibitors with higher selectivity [76]. Another mechanism by which Erk1/2 could positively regulate MC degranulation is suggested by an interesting finding by Pozo-Guisado et al. They demonstrated that Erk1/2 can phosphorylate STIM1, an important calcium sensor in the membrane of the endoplasmic reticulum, and thereby positively modulate store-operated calcium entry, which is mandatory for degranulation to occur [38,77]. Since the p62Dok1-SHIP1 interaction depends on SHIP1 tyrosine phosphorylation [73] and SHIP1 tyrosine phosphorylation is enhanced upon supra-optimal FcεRI triggering [27], Erk1/2 activity could be reduced, contributing to the lack of degranulation under such conditions. Thus, by combining catalytic as well as adaptor functions SHIP1 has the potential to contribute to the regulation of MC activation under supra-optimal Ag conditions in several ways.

As far as other possible negative regulators of supra-optimal FcεRI-induced MC degranulation are concerned, Xiao et al. showed that both SHIP1 and SHP-1 interacted with the FcεRI β-chain and that their phosphorylation was controlled by Lyn [51]. However, studies to date using MCs from different mouse models (*motheaten* (*me*) mice [78] and *viable motheaten* (*me*^*v*^) mice [79]) have not unequivocally confirmed a negative role for SHP-1 with respect to degranulation. Nakata et al. compared BMMCs from wild-type and *me* mice and measured decreased Ca^2+^ mobilization and degranulation in *me* BMMCs in response to Ag [78]. On the other hand, Zhang et al. studied BMMCs from *me*^*v*^ mice, which were found to degranulate in an augmented fashion compared to wild-type BMMCs [79]. The observed differences might be due, in large part, to the diverse effects on protein expression of the different mutations within the gene coding for SHP-1 [80]. Unfortunately, in these SHP-1-related studies, like so many other FcεRI-induced studies to date, Ag dose-responses were not extended to the supra-optimal range.

The Cbl family of E3 ubiquitin-protein ligases comprises three mammalian members, c-cbl, Cbl-b, and Cbl-c. These proteins poly-ubiquitinate various cellular proteins, thereby targeting them for proteasomal degradation [81]. Applying overexpression of c-cbl in the RBL-2H3 MC line, Ota and Samelson previously showed that c-cbl is able to negatively affect MC degranulation by inhibiting the tyrosine kinase Syk [82]. However, a comparison of wild-type and c-cbl-deficient BMMCs did not yield differences in Ag-triggered degranulation [83]. On the other hand, the same study revealed significant differences between wild-type and Cbl-b-deficient BMMCs, indicating non-redundant functions of Cbl family members in MCs. In response to Ag, enhanced tyrosine phosphorylation of the FcεRI ITAMs, Syk, and PLC-γ1/2 as well as Ca^2+^ mobilization was shown in Cbl-b-deficient BMMCs. This resulted in augmented degranulation at every Ag concentration studied with the most prominent effect at high, supra-optimal concentrations [83], suggesting that Cbl-b is involved in the negative control of MC activation in response to supra-optimal cross-linking. Interestingly, CIN85 (Cbl-interacting protein of 85-kDa) was shown to interact with Cbl-b and SHIP1, suggesting the presence of an additional SHIP1-containing inhibitory signalosome ([69,84] & unpublished results). Ag stimulation of RBL-2H3 MCs caused co-translocation of Cbl-b, Lyn, and the FcεRI into lipid rafts [85]. Overexpression of a lipid raft-anchored form of Cbl-b resulted in severe reduction of Ag-triggered tyrosine phosphorylation of the FcεRI ITAMs, Syk, and PLC-γ1/2, Ca^2+^ mobilization, and degranulation [85]. Unexpectedly, degranulation was least affected at high antigen concentrations suggesting differential functions of raft- and non-raft-localized Cbl-b with respect to supra-optimal stimulation. Interestingly, Jnk phosphorylation was almost completely inhibited by the lipid raft-anchored form of Cbl-b, whereas other MAPKs (Erk, p38) were not [85], and Jnk1 was suggested recently to be important for MC degranulation [86]. Again, thorough dose-response studies are lacking for a further evaluation of Jnk function in response to supra-optimal Ag stimulation.

### FcεRI and FcγRIIB (CD32) – brothers in spirit

Immune complex-induced coaggregation of the FcεRI with the inhibitory, low-affinity receptor for IgG, FcγRIIB (CD32), results in suppression of FcεRI-mediated MC activation [87]. In contrast to the FcεRI, FcγRIIB is a single-chain receptor containing extracellular IgG-binding immunoglobulin-like domains, a transmembrane region, and an intracellular domain that contains an immunoreceptor tyrosine-based inhibition motif (ITIM). The tyrosine residue within the ITIM is phosphorylated by Lyn, which is activated via FcεRI cross-linking [88]. Upon ITIM tyrosine phosphorylation, SHIP1 is able to bind to it by means of its SH2-domain, thus contributing to the down-regulation of PI3K-mediated activation events [89,90]. SHIP1 then gets phosphorylated by Lyn as well and is able to recruit a complex consisting of p62Dok1 and RasGAP, thereby negatively regulating the canonical MAPK pathway [72,73]. Interestingly, two crucial elements of FcγRIIB function, Lyn and SHIP1, are central to the suppression of FcεRI signaling at supra-optimal Ag concentrations (this review). Moreover, not only is FcεRI function negatively regulated by submembraneous filamentous actin, but also FcγRIIB-dependent negative regulation is dependent on this cytoskeletal component [91]. Given such similarities it is tempting to suggest that one might be able to transfer mechanisms from one system to the other. PKC-δ could participate in translating FcγRIIB’s negative function and the SHIP1-p62Dok1-RasGAP complex might be involved in supra-optimal FcεRI signaling. In fact, inducible interactions of SHIP1, p62Dok1, and RasGAP can be observed in response to FcεRI triggering (unpublished data). However, one important difference between SHIP1 binding to FcγRIIB vs. FcεRI is that binding of the SHIP1 SH2-domain to the phosphorylated ITIM is of high affinity whereas it is of low affinity to ITAM sequences [66]. Moreover, the FcγRIIB-SHIP1 interaction is stabilized by the small adaptor protein Grb2, which binds to SHIP1 via an SH3-domain and by means of its SH2-domain to a second tyrosine-containing motif within the cytoplasmic domain of FcγRIIB [92]. The reason for this difference in affinity may lie with the distinct actions of SHIP1 on FcεRI- and FcγRIIB-induced signaling. The task of SHIP1 at the FcγRIIB is to “simply” stop cellular activation, quickly and completely. Since SHIP1 is engaged not only in response to supra-optimal triggering of the FcεRI but also during its activation by sub-optimal to optimal Ag concentrations [74], SHIP1’s function here is more an attenuating rather than an inhibitory one. High-affinity binding and attenuation might be mutually exclusive.

### The whole picture (?)

**Figure 3:**
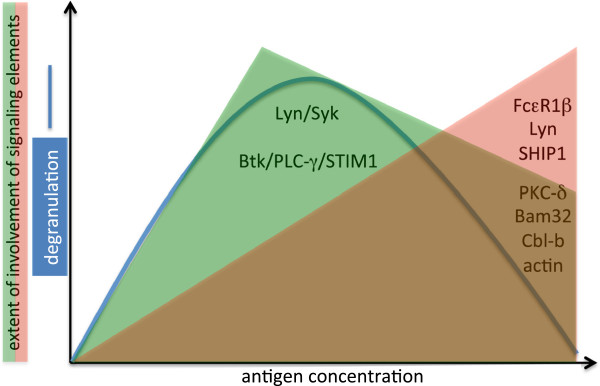
**Activating and inhibitory proteins shape the outcome of FcεRI stimulation by different Ag concentrations.** The Ag-triggered activation of the FcεRI generates both positive and negative signals, which are integrated to determine the quantity of the response. At every Ag concentration both types of signals are generated, however, their ratio and composition differs depending on the antigen dose. Low to optimal Ag concentrations preferentially engage activating signaling elements (green area and proteins indicated there; the tyrosine kinases Lyn and Syk act proximal to the receptor; Btk, PLC-γ, and STIM1 control Ca^2+^ mobilization). In response to high, supra-optimal Ag concentrations negative signaling elements are increasingly recruited (red triangle and proteins indicated there; FcεRIβ, Lyn, and SHIP1 are central to the inhibitory signalosome; PKC-δ, Bam32, Cbl-b, and actin transduce suppressive signals at supra-optimal Ag). Note that the SFK Lyn is both an activating and an attenuating element depending on the Ag dose (see text).

Without a doubt the field has come a long way since the precipitin-type model of supra-optimal FcεRI activation. As stated in this review, several proteins can be assigned inhibitory functions in response to supra-optimal Ag concentrations (Figure 3). However, one can assume that there are more regulatory proteins to be discovered. To accomplish the bell-shaped dose-response curve of FcεRI-mediated MC activation, both positive and negative signals have to be generated and integrated to determine the quantity as well as the quality of the response. Thus, altered positive signaling might result in stronger attenuation at high Ag concentrations. This was shown comparing Ag-triggered histamine release in wild-type, Lyn-deficient, and Btk-deficient BMMCs. Whereas Lyn-deficient BMMCs showed no reduction of degranulation at high Ag concentrations, compared to wild-type cells, Btk-deficient BMMCs exerted stronger attenuation than wild-type BMMCs [93], consistent with the accepted positive regulatory role for Btk in FcεRI signaling [94]. A further interesting point is the differential dose-response behavior of Ag-triggered MCs with respect to different pro-inflammatory effector functions. Both immediate degranulation and subsequent production of pro-inflammatory cytokines (e.g. IL-6 and TNF-alpha follow a bell shape. However, the descending part of the curve is significantly steeper concerning cytokine production [27,28,95]. Moreover, Rivera and coworkers elegantly demonstrated that Ag-induced mediators, such as various cytokines and chemokines, needed different Ag concentrations for their optimal expression [96]. This strongly suggests that there is not one activating and one inhibitory signal, but for different MC responses there might be specially balanced signaling pathways or networks.

## Conclusions

The bell-shaped dose-response behavior represents a remarkable feature of the FcεRI. Nature provides us with an efficient shut-off mechanism at high, supra-optimal Ag concentrations, which could be employed to alleviate allergic symptoms. By deciphering the molecular language of inhibitory signaling in response to supra-optimal Ag concentrations one might identify novel targets for pharmacologic therapeutic interventions to benefit patients with acute and chronic allergic diseases. A deeper understanding of this mechanism(s) should also help to elucidate the molecular events that occur during hyposensitization of allergy patients. Finally, the bell-shaped dose response pattern is not unique to the FcεRI, but can also be found with other receptors that signal in an ITAM-dependent manner, such as the FcγRIII (CD16) and the B cell antigen receptor [97,98]. Thus, improving our understanding of the regulation of FcεRI signaling will also enhance our understanding of other immunologically important receptors.

## Competing interests

The author declares that he has no competing interests.
